# Differential association between physical activity behaviours and dynapenia by comorbid diseases in community-dwelling Korean older adults

**DOI:** 10.1186/s11556-024-00340-z

**Published:** 2024-03-08

**Authors:** Jae Hyeon Park, Hyung Seok Nam, Mina Park, Yeo Hyung Kim

**Affiliations:** 1 Department of Rehabilitation Medicine, Hanyang University College of Medicine, Seoul, Republic of Korea; 2 Department of Rehabilitation Medicine, Sheikh Khalifa Specialty Hospital, Ras Al Khaimah, UAE; 3 Department of Rehabilitation Medicine, College of Medicine, The Catholic University of Korea, Seoul, 06591 Republic of Korea

**Keywords:** Aged, Exercise, Noncommunicable diseases, Sarcopenia, Sedentary behaviour

## Abstract

**Background:**

Physical activity (PA) behaviours and comorbid diseases are associated with muscle strength. However, the association between dynapenia and detailed PA behaviours, including participation in aerobic and resistance exercises and sedentary behaviour (SB), in relation to comorbid diseases has not yet been investigated. Using nationwide data, this study aimed to evaluate the independent association of dynapenia with detailed PA behaviour (participation in aerobic and resistance exercises and SB), and assess the differential associations of detailed PA behaviour with dynapenia according to comorbid diseases with prevalent sarcopenia.

**Methods:**

A total of 7,558 community-dwelling older adults aged ≥ 65 years who were included in the Korea National Health and Nutrition Examination Survey from 2014 to 2019 were included in the present study. Cross-sectional associations between PA behaviours (participation in aerobic exercise, participation in resistance exercise, and SB) and dynapenia were analysed using complex-sample multivariable-adjusted logistic regression models according to the type of comorbid disease (cardiovascular disease [CVD], diabetes mellitus [DM], and chronic lung disease [CLD]).

**Results:**

Sufficient aerobic exercise, sufficient resistance exercise, and low sedentary time of < 420 min/day showed independent negative associations with dynapenia (odds ratio [OR], 0.71; 95% confidence interval [CI], 0.60–0.83; OR, 0.54; 95% CI, 0.42–0.69; and OR, 0.84; 95% CI, 0.72–0.97, respectively). Among the participants with CVD or CLD, the associations of sufficient resistance exercise (OR, 0.46; 95% CI, 0.26–0.82 and OR, 0.51; 95% CI, 0.35–0.75 for CVD and CLD, respectively) and low sedentary time (OR, 0.66; 95% CI, 0.45–0.98 and OR, 0.71; 95% CI, 0.55–0.93 for CVD and CLD, respectively) with dynapenia were significant, whereas the association of sufficient aerobic exercise with dynapenia was insignificant. Meanwhile, in participants with DM, sufficient aerobic exercise (OR, 0.70; 95% CI, 0.52–0.94) and sufficient resistance exercise (OR, 0.45; 95% CI, 0.29–0.70) were independently associated with dynapenia, whereas no association between SB and dynapenia was found.

**Conclusion:**

We observed an independent inverse association between PA behaviours and dynapenia. Disease-specific associations between each PA behaviour (sufficient aerobic exercise, sufficient resistance exercise, and low sedentary time) and dynapenia differed in the older adults. Therefore, these differences should be acknowledged during interventions for this population.

## Introduction

Dynapenia is defined as an age-related decline in muscle strength, while sarcopenia is defined as the loss of both skeletal muscle mass and muscular strength with age [1, 2]. Dynapenia has recently gained increased attention because muscle strength has been demonstrated to be more clinically important in physical function and mortality compared with muscle mass in older populations [2, 3]. Furthermore, dynapenia can be diagnosed more easily by assessing muscle strength using a hand-held dynamometer, while the diagnosis of sarcopenia requires the measurement of muscle strength, muscle mass, and function [4]. Therefore, the Asian Working Group for Sarcopenia recommends prioritizing the assessment of muscle strength as a primary measure for the early identification of older adults with sarcopenia or those at risk of sarcopenia, without the use of advanced diagnostic devices [1]. Early identification and intervention for decline in muscle strength are crucial for clinicians because dynapenia causes functional impairment, physical disability, and increased all-cause mortality [1, 4, 5]. Furthermore, dynapenia contributes to a deterioration of quality of life in older adults through the decline in physical health and functional impairment, as well as social isolation exacerbated by physical disability [4, 6].

Muscle strength in the elderly is associated with multiple factors including behavioural factors, and comorbid diseases [7]. Physical activity (PA) behaviour is a well-known behavioural factor related to muscle strength [7, 8]. PA behaviour has several different dimensions including the type (aerobic and resistance) of PA, the intensity of PA (light, moderate and vigorous) and sedentary behaviour (SB). Previous studies have reported that higher PA levels (including total PA, moderate to vigorous PA, and light PA) as well as reduced SB (achieved by reducing sitting, reclining, and lying time) is associated with greater independent hand grip strength in older adults [8, 9]. However, research investigating the associations between dynapenia and detailed PA behaviour (including aerobic exercise, anaerobic exercise, and SB) is lacking, despite the importance of identifying specific PA behaviours to plan interventions to cope with dynapenia.

Comorbid diseases are also important factors related with muscle strength in older adults. Several chronic diseases in older adults share a common pathogenesis with dynapenia and can adversely impact or be impacted by dynapenia [10]. Cardiovascular disease (CVD), diabetes mellitus (DM) and chronic lung disease (CLD) are common representative comorbid diseases in older adults, which are associated with a decline in muscle strength and share risk factors with dynapenia [10, 11]. Furthermore, physical inactivity is a major risk and prognostic factor for dynapenia and the representative comorbid diseases in older adults [11]. Thus, studies have assessed the association between PA intensity and muscle strength in individuals with CVD, DM, or CLDs [12–14]. However, the results according to comorbid diseases have been inconsistent as follows: PA level and SB was not associated with grip strength in patients with DM [13], PA level was significantly lower in cardiac patients with sarcopenia than in those without sarcopenia [12], and PA level is inversely correlated with dynapenia in older adults with chronic obstructive pulmonary disease (COPD) [14]. Furthermore, those studies only investigated the association between PA level and muscle strength or sacopenia in specific disease populations. The studies did not evaluate the association between PA level and dynapenia across comorbid diseases within the same large population nor examine the relationship between the type of PA and muscle strength.

Therefore, this study aimed to 1) evaluate the independent association of dynapenia with detailed PA behaviours (participation in aerobic and resistance exercises and SB), adjusted for possible confounders and 2) assess the associations of detailed PA behaviours with dynapenia stratified by comorbid diseases (CVD, DM, and CLD), which are well-known to be associated with a high prevalence of dynapenia, using nationwide representative data.

## Methods

### Study sample

The study population was drawn from the Korea National Health and Nutrition Examination Survey (KNHANES) VI, VII, and VIII. The KNHANES is conducted by the Korea Disease Control and Prevention Agency and contains structured health interviews and examinations conducted at mobile centres. To collect data representing all non-institutionalised Korean citizens, the KNHANES sampling plan follows a multistage cluster probability design. Once the survey area and households were randomly selected, the eligible individuals within those households were invited to participate by household visits, mails, and calls. A specialized survey team visited the area using a mobile examination vehicle to conduct medical examinations and health surveys. These mobile centres are equipped with medical and examination facilities, allowing participants to undergo anthropometric measurements, blood and urine samplings, eye and dental examinations, and other health-related assessments Anonymised raw data and instructions for analysis are accessible to the public on the KNHANES website (https://knhanes.kdca.go.kr/knhanes). All the KNHANES participants provided informed consent.

The inclusion criteria for the current study consisted of older adults aged 65 years or older who completed the questionnaires on physical activity and handgrip measurements. From 2014 to 2019, a total of 47,309 Koreans participated in the KNHANES. After excluding 37,484 participants aged below 65 years, 9,825 older adults aged 65 years or older (men, *n* = 4240, 43.16%; women, *n* = 5585, 56.84%) were included. Although the exact reasons for missing values are not reported on an individualized basis, participants could refuse to answer the physical activity questionnaires or the handgrip strength measurement. There were also cases of incomplete responses to the physical activity questionnaires. The handgrip strength of individuals with amputations of, paralysis of, or braces on the upper extremities was not measured. Those who had undergone wrist or hand surgeries within 3 months and with wrist or hand pain within 7 days prior to the KNHANES survey were also excluded from the handgrip strength measurement. After further excluding participants with missing data for physical activity (*n* = 1,213) or handgrip strength (*n* = 1,207), a total of 7,558 (men, *n* = 3,419, 45.24%; women, *n* = 4,139, 54.76%) older adults were included in the analysis.

Individuals without a history of the four major noncommunicable diseases (CVD, DM, CLD, and cancers) documented by the World Health Organization (WHO) were defined as participants without a major noncommunicable diseases [15]. Since the current study focused on CVD, DM, and CLD [11, 15], participants with these conditions were categorized into subgroups. Individuals with more than one noncommunicable disease (CVD, DM, or CLD) were included in more than one subgroup.

The Institutional Review Board of Uijeongbu St. Mary’s Hospital, Republic of Korea waived the need for ethical approval for this study because publicly available data were analysed.

### Handgrip strength assessment

In the KNHANES, the handgrip strength of both hands was measured in participants using a digital dynamometer (TKK 5401; Takei Scientific Instruments Co., Ltd., Niigata, Japan). The handgrip strength of each hand, starting with the dominant hand, was measured three times, with a 60-s rest between measurements. Measurements were performed in the standing position with the arms naturally lowered and the elbows and wrists extended.

The highest of the six handgrip measurement values was considered as the final handgrip strength of the participant [16]. In accordance with the Asian Working Group for Sarcopenia 2019 Consensus, dynapenia was defined using diagnostic cutoffs of handgrip strength, which is < 28.0 kg for men and < 18.0 kg for women [1].

### Physical activity evaluation

Participation in aerobic exercise and SB were assessed using a validated Korean version of the WHO Global PA Questionnaire (GPAQ) [17, 18]. The GPAQ assesses the frequency (days/week) and duration (min/day) of participating in moderate and vigorous aerobic physical activities within a typical week that increases ones breathing or heart rate. Weekly time spent in moderate and vigorous aerobic exercises (min/week) was calculated from these frequency and duration variables, according to the GPAQ analysis guide [18]. The question 16 of the GPAG evaluates the time usually spent sitting or reclining on a typical day (min/day). Because the data on resistance exercise is not obtainable by the GPAQ, the frequency (days/week) of participation in resistance exercises such as push-ups, sit-ups, dumbbells, weights, or barbells in the last week was separately surveyed.

The obtained aerobic exercise and resistance exercise levels were categorised into binary variables (sufficient and insufficient) based on the WHO global recommendations on physical activity for health [19]. Sufficient aerobic exercise was defined as ≥ 75 min/week of vigorous-intensity aerobic physical activity, ≥ 150 min/week of moderate-intensity aerobic physical activity, or an equivalent combination of moderate- and vigorous-intensity activity according to the WHO global recommendations on physical activity for health [19]. Participants who did not meet these criteria for sufficient aerobic exercise were categorised as insufficient aerobic exercise. According to the WHO global recommendations on physical activity for health, resistance exercise ≥ 2 days/week was categorised as sufficient resistance exercise [19]. Individuals who performed resistance exercise < 2 days/week were classified as insufficient resistance exercise. Participants were categorized as ‘high sedentary time’ if they had ≥ 420 min of daily sedentary time and as ‘low sedentary time’ if they had < 420 min/day [20].

### Comorbidities

Participants diagnosed with stroke, angina, or myocardial infarction by a doctor were categorised as having a CVD [21]. Older adults with DM were defined as those diagnosed with DM by a doctor, taking hypoglycaemic agents or insulin injections, with an HbA1c ≥ 6.5%, or with a fasting blood glucose ≥ 126 mg/dL. Individuals diagnosed with chronic obstructive pulmonary disease or asthma by a doctor, with a forced expiratory volume in 1 s (FEV1)/forced vital capacity (FVC) of < 0.7, or an FVC < 80% of predicted value on spirometry were categorised as having CLD. History of depression and cancer diagnosed by a doctor was also recorded.

### Covariates

Age, sex, and body mass index (BMI) were considered essential covariates due to their well-established associations with physical activity and handgrip strength [22–24]. After participants were asked to remove their shoes and stand upright against a stadiometer, the survey staff measured their height to the nearest mm. The staff recorded weight to the nearest 0.1 kg after the participants removed their heavy clothing and stood on a calibrated scale. Then BMI was obtained using the following formula: weight (kg) / height (m^2^). Age was calculated using the birth date of the participants.

Other covariates (alcohol, smoking, occupation, residence, and education) were selected based on their proposed associations with physical activity and handgrip strength in previous studies [25–28] and their clinical significance. Alcohol consumption data was collected through self-reported information regarding the frequency and quantity of alcohol consumption. Using this information, the average daily consumption of alcohol for each individual was calculated, as previously reported [29]. Participants were categorised as excessive alcohol drinkers if they consumed over 10 g (women) or 20 g (men) of alcohol per day. Information on smoking behaviour (never, past, or current), occupational status (yes or no), area of residence (urban or rural), and level of education (middle school or lower or high school or higher) was self-reported by participants during the interview.

### Statistical analysis

The characteristics of study participants were evaluated using complex sample descriptive statistics. The weighted prevalence of dynapenia according to PA behavioural status was compared using a complex sample chi-square test. The association of participation in aerobic and resistance exercises and SB with dynapenia was analysed using complex sample multivariable-adjusted logistic regression models. To assess the potential confounding associations, four models were sequentially constructed. Furthermore, to evaluate the disease-specific association between PA and dynapenia, sequential logistic regression models were built separately for participants with CVD, DM, and CLD and participants without a major noncommunicable disease. To reduce the bias from missing data, unequal sampling, and non-response, we applied sampling weights considering the multistage cluster probability sampling design. We used complex-sample procedures of SPSS (version 24; IBM/SPSS Inc., Armonk, NY, USA).

## Results

### Participant characteristics

The weighted mean age of the included 7,558 participants was 72.45 years (standard error, 0.08). Among the total study participants, the weighted prevalence of participants without a major noncommunicable disease was 38.64%, with CVD was 13.87%, with DM was 28.91%, and with CLD was 32.31%.

The characteristics of the participants in each group are presented in Table 1. The weighted prevalence of dynapenia was 24.76% (all participants), 24.28% (without a major noncommunicable disease), 30.46% (CVD), 28.95% (DM), and 20.10% (CLD). The weighted prevalence of older adults who engaged in insufficient aerobic exercise was 66.84% (all participants), 66.91% (without a major noncommunicable disease), 72.25% (CVD), 69.24% (DM), and 63.30% (CLD). The weighted prevalence of older adults who engaged in insufficient resistance exercise were 81.44% (all participants), 82.06% (without a major noncommunicable disease), 84.28% (CVD), 84.30% (DM), and 77.51% (CLD). Among the participants, 56.43% (all participants), 53.42% (without a major noncommunicable disease), 65.16% (CVD), 60.40% (DM), and 56.05% (CLD) had high sedentary time.

**Table 1:** Characteristics of study participants

	All participants	Participants without a major NCD	Cardiovascular disease	Diabetes mellitus	Chronic lung disease
Unweighted number (weighted %)	7558 (100.00)	2908 (38.64)	1070 (13.87)	2192 (28.91)	2481 (32.31)
Age (years)	72.45 ± 0.08	71.88 ± 0.12	73.63 ± 0.17	72.98 ± 0.13	72.50 ± 0.12
Body mass index (kg/m^2^)	24.19 ± 0.04	23.93 ± 0.07	24.51 ± 0.11	24.79 ± 0.08	24.35 ± 0.07
Female sex (%)	55.31 (0.61)	66.00 (0.98)	47.93 (1.71)	54.44 (1.20)	39.02 (1.05)
Excessive alcohol (%)	6.44 (0.33)	6.11 (0.51)	4.56 (0.72)	7.00 (0.64)	8.63 (0.67)
Smoking (%)					
Never	61.52 (0.62)	71.01 (0.99)	53.52 (1.66)	60.02 (1.19)	47.72 (1.07)
Past	28.97 (0.57)	21.91 (0.88)	35.94 (1.61)	29.78 (1.12)	38.74 (1.07)
Current	9.51 (0.42)	7.08 (0.65)	10.54 (1.06)	10.20 (0.73)	13.54 (0.79)
Having an occupation (%)	33.27 (0.74)	35.03 (1.12)	26.53 (1.62)	30.77 (1.23)	36.00 (1.17)
Residence (%)					
Urban	76.40 (1.61)	76.09 (1.86)	76.34 (2.01)	76.86 (1.71)	76.33 (1.80)
Rural	23.60 (1.61)	23.91 (1.86)	23.66 (2.01)	23.14 (1.71)	23.67 (1.80)
Education (%)					
Middle school or lower	71.82 (0.81)	73.48 (1.17)	73.60 (1.63)	74.28 (1.16)	67.17 (1.22)
High school or higher	28.18 (0.81)	26.52 (1.17)	26.40 (1.63)	25.72 (1.16)	32.83 (1.22)
Depression (%)	6.42 (0.33)	6.74 (0.57)	8.34 (0.92)	6.82 (0.62)	5.07 (0.49)
Cancer (%)	10.46 (0.41)	0.00 (0.00)	9.09 (1.06)	10.49 (0.76)	9.51 (0.65)

Data are presented as mean ± standard error or percentage (standard error)

*NCDs* noncommunicable diseases

### Association of physical activity behaviours with dynapenia

**Fig. 1:**
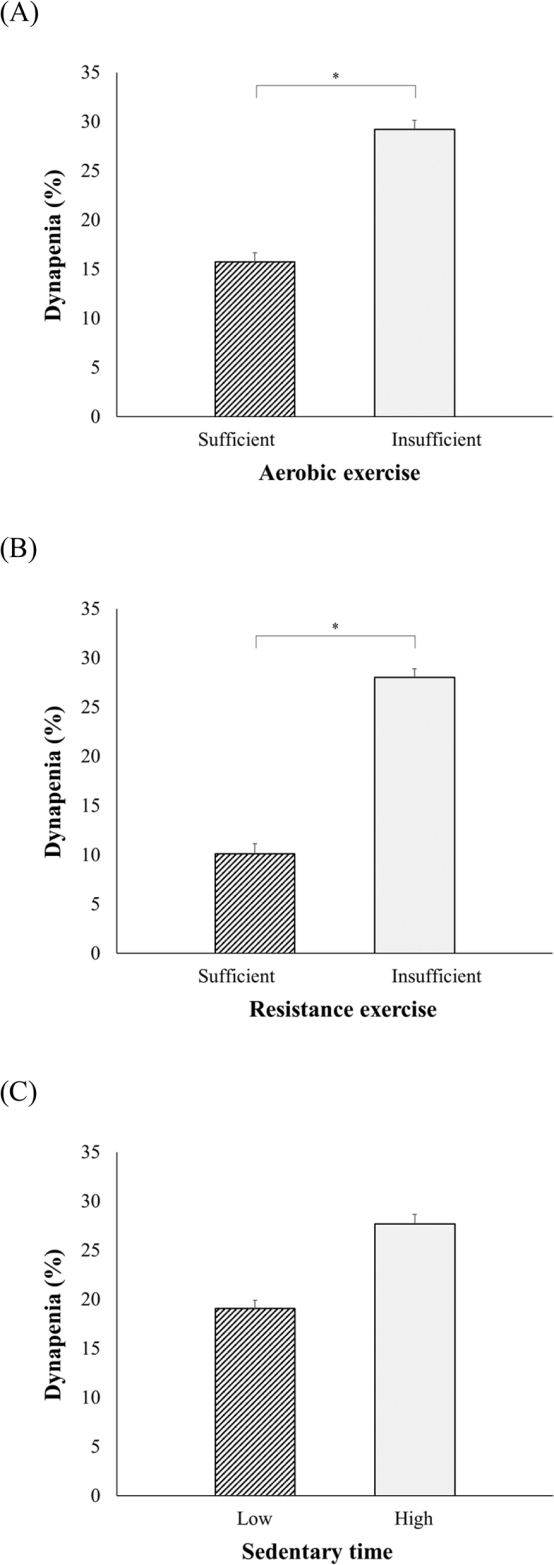
Weighted prevalence of dynapenia according to each PA behaviour. **A** aerobic exercise, **B** resistance exercise, and (**C**) sedentary behaviour

The weighted prevalence of dynapenia according to each PA behaviour level is shown in Fig. 1A, B, and C. The weighted prevalence of dynapenia was higher in participants who performed insufficient aerobic exercise than in those who performed sufficient aerobic exercise (29.23% vs. 15.74%, *P* < 0.001), in participants who performed insufficient resistance exercise than in those who performed sufficient resistance exercise (28.04% vs. 10.14%, *P* < 0.001), and in participants with high sedentary time than in participants with low sedentary time (27.70% vs. 19.07%, *P* < 0.001).

Table 2 presents the negative associations of the levels of aerobic exercise, resistance exercise, and SB with dynapenia in older adults. Sufficient aerobic exercise compared with insufficient aerobic exercise was significantly associated with a lower likelihood of dynapenia (adjusted odds ratio [OR], 0.71; 95% confidence interval [CI], 0.60–0.83; in Model 4, fully adjusted) independent of multiple sociodemographic variables, comorbidities, resistance exercise, and SB. Sufficient resistance exercise compared with insufficient resistance exercise was related with significantly lower odds of dynapenia (adjusted OR, 0.54; 95% CI, 0.42–0.69; in Model 4) after adjusting for sociodemographic variables, comorbidities, aerobic exercise, and SB. The participants with low sedentary time showed a 16% lower odds of dynapenia than that shown by participants with high sedentary time (adjusted OR, 0.84; 95% CI, 0.72–0.97; in Model 4) after adjusting for multiple confounders.

**Table 2:** Association of the level of aerobic and resistance exercises and sedentary behaviour with dynapenia

	Model 1	Model 2	Model 3	Model 4
Aerobic exercise^a^				
Insufficient	1.00	1.00	1.00	1.00
Sufficient	0.58 (0.50–0.68)	0.62 (0.53–0.73)	0.65 (0.56–0.77)	0.71 (0.60–0.83)
*P* value	< 0.001	< 0.001	< 0.001	< 0.001
Resistance exercise^b^				
Insufficient	1.00	1.00	1.00	1.00
Sufficient	0.44 (0.35–0.56)	0.49 (0.38–0.62)	0.51 (0.40–0.66)	0.54 (0.42–0.69)
*P* value	< 0.001	< 0.001	< 0.001	< 0.001
Sedentary time^c^				
High	1.00	1.00	1.00	1.00
Low	0.80 (0.70–0.92)	0.79 (0.69–0.91)	0.80 (0.69–0.92)	0.84 (0.72–0.97)
*P* value	0.002	0.001	0.002	0.016

Data are presented as adjusted odds ratios (95% confidence interval)

Model 1: adjusted for age, sex, and body mass index

Model 2: Model 1 was further adjusted for alcohol consumption, smoking status, occupation, residence, education, depression status, and cancer status

Model 3: Model 2 was further adjusted for cardiovascular disease, diabetes, and chronic lung disease

^a^Model 4: Model 3 was further adjusted for participation in resistance exercise and sedentary behaviour

^b^Model 4: Model 3 was further adjusted for participation in aerobic exercise and sedentary behaviour

^c^Model 4: Model 3 was further adjusted for participation in aerobic and resistance exercises

### Association of physical activity behaviours with dynapenia by comorbid disease

The associations of PA and SB with dynapenia by comorbid disease are shown in Table 3. Among the participants with CVD or CLD, significant negative associations of the levels of aerobic and resistance exercises and SB with dynapenia were observed, before adjusting for confounders (Model 1). The negative associations of dynapenia with the level of resistance exercise (adjusted OR, 0.46 and 0.51 in Model 4 of CVD and CLD, respectively) and SB (adjusted OR, 0.66 and 0.71; in Model 4 of CVD and CLD, respectively) remained significant after adjusting for multiple confounders. However, the association between the level of aerobic exercise and dynapenia became insignificant after adjusting for the level of resistance exercise and SB.

**Table 3:** Association of aerobic and resistance exercises and sedentary behaviour levels with dynapenia by comorbid disease

	Cardiovascular disease	Diabetes mellitus	Chronic lung disease
Sufficient aerobic exercise^a^			
Model 1	0.55 (0.38–0.81)	0.59 (0.45–0.78)	0.69 (0.53–0.90)
Model 2	0.58 (0.39–0.86)	0.61 (0.46–0.80)	0.72 (0.55–0.95)
Model 3	0.64 (0.42–0.97)	0.64 (0.48–0.85)	0.74 (0.56–0.97)
Model 4	0.73 (0.48–1.12)	0.70 (0.52–0.94)	0.83 (0.62–1.09)
Sufficient resistance exercise^b^			
Model 1	0.43 (0.25–0.72)	0.42 (0.28–0.64)	0.43 (0.29–0.64)
Model 2	0.43 (0.25–0.74)	0.43 (0.28–0.65)	0.48 (0.33–0.72)
Model 3	0.43 (0.24–0.76)	0.47 (0.31–0.71)	0.49 (0.33–0.72)
Model 4	0.46 (0.26–0.82)	0.45 (0.29–0.70)	0.51 (0.35–0.75)
Low sedentary time^c^			
Model 1	0.66 (0.47–0.92)	0.85 (0.66–1.09)	0.71 (0.55–0.92)
Model 2	0.65 (0.46–0.92)	0.86 (0.67–1.11)	0.69 (0.53–0.89)
Model 3	0.63 (0.43–0.92)	0.88 (0.68–1.14)	0.69 (0.54–0.90)
Model 4	0.66 (0.45–0.98)	0.91 (0.70–1.19)	0.71 (0.55–0.93)

Data are presented as adjusted odds ratios (95% confidence interval)

Model 1: adjusted for age, sex, and body mass index

Model 2: Model 1 was further adjusted for alcohol consumption, smoking status, occupation, residence, education, depression status, and cancer status

Model 3: Model 2 was further adjusted for any two of the comorbid diseases including cardiovascular disease, diabetes, and chronic lung disease

^a^Model 4: Model 3 was further adjusted for participation in resistance exercise and sedentary behaviour

^b^Model 4: Model 3 was further adjusted for participation in aerobic exercise and sedentary behaviour

^c^Model 4: Model 3 was further adjusted for participation in aerobic and resistance exercises

Meanwhile, participants with DM showed different patterns of association of PA and SB with dynapenia compared with that showed by those with CVD or CLD. Sufficient levels of aerobic and resistance exercises were associated with lower odds of dynapenia before adjusting for confounders (Model 1) and after sequential adjustments for confounders (Models 2, 3, and 4). In the fully adjusted models (Model 4), sufficient levels of aerobic and resistance exercises were associated with a 0.70-fold and 0.45-fold lower odds of dynapenia than that observed with insufficient levels of aerobic and resistance exercises, respectively. In contrast, the level of SB among participants with DM was not associated with dynapenia.

## Discussion

It is imperative to assess the association between dynapenia and detailed PA behaviour after adjusting for other PA behaviours. This is essential for identifying modifiable risk factors of dynapenia, given the potential interrelatedness of PA behaviours. In this study, an inverse relationship was observed between dynapenia and recommended PA behaviours (sufficient aerobic exercise, sufficient resistance exercise, and low sedentary time) after adjusting for multiple confounding factors. Among the PA behaviours, patients who performed sufficient resistance exercise had lower odds of developing dynapenia than that had by those who performed sufficient aerobic exercise and those with non-SB. Furthermore, the relationship between detailed PA behaviours and dynapenia was different according to the type of comorbid disease after adjusting for confounders. In participants with CVD and CLD, higher levels of resistance exercise and non-SB were independently associated with a lower likelihood of dynapenia. In contrast, sufficient aerobic and resistance exercises were inversely associated with dynapenia in older adults with DM.

Physical inactivity is a fundamental behavioural risk factor for sarcopenia, and higher levels of PA are associated with higher muscle strength [8]. Therefore, engaging in regular PA, including aerobic and resistance exercises, is recommended for older adults [1, 19]. However, the effects of aerobic exercise on muscle strength are still controversial, although several studies have reported the association between aerobic PA levels and muscle strength [8]. Some previous studies have demonstrated that aerobic exercise or aerobic PA level was not associated with muscle strength [13, 30]. Furthermore, it is uncertain whether most aerobic exercises that primarily engage the lower extremity muscles, such as walking and running, could enhance hand grip strength. In this study, sufficient PA including aerobic and resistance exercises was associated with a lower likelihood of dynapenia. In particular, we found that sufficient resistance exercise had the lowest odds of dynapenia compared with that had by sufficient aerobic exercise and non-SB. The results of our study are consistent with those of previous reports showing that resistance exercise most effectively preserves and improves muscle strength in older adults [31]. We also found that sufficient aerobic exercise had an independent lower odds of dynapenia, even after adjusting for the level of resistance exercise and other confounders. Previous studies have reported that aerobic exercise alone can improve muscle function and mass [32, 33].

Our results revealed that after adjusting for PA behaviours and other possible confounding factors, SB was independently associated with dynapenia in older adults, which is consistent with a previous meta-analysis [8]. However, SB had a lower effect estimate for dynapenia than did aerobic and resistance exercises. Waking behaviours such as sitting, lying down, or reclining with an energy expenditure of ≤ 1.5 metabolic equivalents are considered SBs [34]. Based on our definition of aerobic exercise as moderate-to-vigorous intensity aerobic PA, a decrease in SB does not imply an increase in aerobic exercise [18, 19]. Therefore, the relatively weak association between SB and dynapenia observed in our study may be attributed to the increase in light-intensity PA (rather than an increase in moderate to vigorous PA), which reduced SB in older adults. The results regarding the effects of replacing SB with light-intensity PA on muscle strength in older adults are controversial. In one study, the substitution of SB with one hour of light-intensity PA per day resulted in increased grip strength [35]. However, in other studies, this replacement did not lead to significant changes in the grip strength [36, 37].

In the stratified analyses according to comorbid diseases, participants with CLD exhibited relatively more active PA behaviour, engaged in sufficient aerobic and resistance exercise, and exhibited less sedentary behaviour than those observed with CVD or DM. These results may be due to the criteria used to diagnose comorbid diseases in the KHANES, and it appears that participants with mild symptoms who had no or mild exercise intolerance may have been included as part of participants with CLD. However, the patterns of association between detailed PA behaviours and dynapenia was similar in participants with CVD and CLD, while participants with DM exhibited a different pattern. The significant negative association between sufficient aerobic exercise and dynapenia became insignificant after adjusting for sufficient resistance exercise and SB levels in participants with CVD and CLD; however, this association remained significant in those with DM. These results may be due to shared disease characteristics between CVD and CLD, such as exercise intolerance and mechanical inefficiency [38]. In previous studies, aerobic PA level was reported to be inversely correlated with muscle strength or sarcopenia in participants with CVD [12] and COPD [14], which was consistent with our results. However, Mickute et al. [13] reported that aerobic PA level was not associated with dynapenia in patients with DM. However, those previous studies did not adjust for resistance exercise levels when they investigated the association between PA and sarcopenia or dynapenia or evaluate the association across comorbid diseases within the same nationwide large population.

Several explanations for the non-significant association observed between aerobic exercise and dynapenia after adjusting for other PA behaviours in participants with CVD or CLD have been proposed. First, aerobic exercise may not have been sufficient to improve the muscle strength of these participants. The proposed mechanisms for the effectiveness of aerobic exercise on muscle strength or mass are the reduction of reactive oxygen species by mitochondrial energetics and improvement of muscle protein metabolism [33]. Previous studies have reported that patients with CVD or CLD exhibit mitochondrial dysfunction and impaired protein metabolism [39–41]. However, as mitochondrial dysfunction and protein metabolism abnormalities have also been reported in patients with DM [42], the underlying mechanisms for the differences between DM and other diseases remain unclear. Second, older adults with CVD or CLD may experience difficulty performing sufficient aerobic exercise, even if they do not have muscle weakness or dynapenia. Furthermore, patients with CVD or CLD are often intolerant to aerobic exercise [11].

Interestingly, SB was not significantly associated with dynapenia in older adults with DM but was significantly associated with CVD and CLD. The association between SB and dynapenia in patients with DM has not been thoroughly investigated. Li et al. reported an inverse correlation between SB and appendicular skeletal muscle index in patients with DM [43]. Mickute et al. found that patients with DM who have impaired physical function (measured by the short physical performance battery) did not have significantly different total sedentary time compared with that had by patients with DM who had normal physical function. However, prolonged SB (continuous SB ≥ 30 min) was longer in the impaired physical function group than in the normal function group [13]. In our study, SB was assessed using total sedentary time, and prolonged SB was not evaluated. This could have resulted in the lack of a significant association between SB and dynapenia. Breaking up prolonged SB with bouts of light-intensity PA improves glucose control in patients with DM [44]. Further studies are needed to investigate the effects of total sedentary time, prolonged SB, and the substitution of SB with light-intensity PA on dynapenia in patients with DM.

The strength of our study is the investigation of the association between detailed PA behaviour and dynapenia, with adjustments for various possible confounders, using large, nationally representative data. Furthermore, we performed analyses stratified by comorbid diseases with high sarcopenia prevalence. To the best of our knowledge, this study is the first attempt to evaluate the association between dynapenia and detailed PA behaviours, stratified by comorbid diseases, using nationwide representative data. However, this study has several limitations. First, as this study used a cross-sectional design, we could not determine cause-effect relationships. Second, although the GPAQ is a valid and reliable tool for assessing PA behaviour, detailed PA levels were not objectively evaluated using instruments such as accelerometers or pedometers. Although objective tools can provide a more precise and detailed assessment of physical activity levels, practical problems associated with participant compliance, cost, privacy issues, and data processing requirements, may exist in previously published population-based studies. Third, because the physical activity information collected in the KNHANES includes aerobic exercise, resistance exercise, and sedentary behaviour, the association of the other types of exercise, such as multi-component exercise, with dynapenia could not be assessed in this study. Furthermore, the dichotomous categorisation of exercise levels based on the WHO recommendations may hinder the uncovering of important information. Fourth, the severity of comorbid diseases or medications taken by participants may have influenced the results; however, such detailed information could not be obtained. Although nutrition-related factors may affect physical activity and handgrip strength, these factors were not considered in the current study. Finally, since individuals with more than one of the three morbidities (CVD, DM, or CLD; those with multimorbidity) were included in more than one subgroup, the analyses to compare subgroup characteristics (CVD vs DM vs CLD) was impossible. Thus, future studies are needed to objectively investigate PA levels using a device, examine the type and duration of resistance exercise, and assess nutritional status through a longitudinal study.

## Conclusions

Sufficient aerobic and resistance exercises and a low sedentary time were independently and inversely associated with dynapenia in community-dwelling older adults. Furthermore, the association between each detailed PA behaviour and dynapenia varied depending on the comorbid disease. Comorbid diseases of older adults should be considered when investigating the relationship between each PA behaviour and dynapenia. The assumption of a simple negative association between PA and dynapenia is not valid without considering the type and intensity of PA, SB, and comorbid diseases.

## Data Availability

Anonymised raw data are accessible to the public on the KNHANES website (https://knhanes.kdca.go.kr/knhanes).

## Abbreviations

CVD: Cardiovascular disease
DM: Diabetes mellitus
PA: Physical activity
SB: Sedentary behaviour
GPAQ: Global PA Questionnaire
KNHANES: Korea National Health and Nutrition Examination Survey
FEV1: Forced expiratory volume in 1 s
FVC: Forced vital capacity
OR: Odds ratio
CI: Confidence interval
